# Low Serum IGF-1 in Boys with Recent Onset of Juvenile Idiopathic Arthritis

**DOI:** 10.1155/2018/3856897

**Published:** 2018-11-28

**Authors:** Anna-Carin Lundell, Malin Erlandsson, Maria Bokarewa, Hille Liivamägi, Karin Uibo, Sirje Tarraste, Tiina Rebane, Tiina Talvik, Chris Pruunsild, Rille Pullerits

**Affiliations:** ^1^Department of Rheumatology and Inflammation Research, Institute of Medicine, Sahlgrenska Academy at University of Gothenburg, Sweden; ^2^Children's Clinic, Tartu University Hospital, Tartu, Estonia; ^3^Tallinn Children's Hospital, Tallinn, Estonia; ^4^Institute of Clinical Medicine, Faculty of Medicine, Tartu University, Tartu, Estonia; ^5^Department of Clinical Immunology and Transfusion Medicine, Sahlgrenska University Hospital, Gothenburg, Sweden

## Abstract

**Background:**

Liver-derived insulin-like growth factor-1 (IGF-1) contributes bone formation. Decreased IGF-1 levels are common in juvenile idiopathic arthritis (JIA), but whether IGF-1 is related to sex and differ during the pathogenic progress of JIA is unknown.

**Objective:**

The aim of this study was to examine IGF-1 levels in boys and girls with newly diagnosed JIA, with established JIA and in controls.

**Methods:**

The study group included 131 patients from the Estonian population-based prevalence JIA study. Blood samples were obtained from 27 boys and 38 girls with early JIA (≤1 month from the diagnosis), 29 boys and 36 girls with established JIA (mean disease duration 18 months), and from 47 age- and sex-matched controls.

**Results:**

IGF-1 levels in boys were significantly decreased in early JIA compared to male controls, while IGF-1 levels in girls were comparable between JIA and controls. In early JIA, IGF-1 levels were 12-fold lower in boys relative to girls. In controls, IGF-1 levels correlated with both age and height, while these correlations were lost in boys with early JIA.

**Conclusion:**

We report a sex-dependent deficiency in serum IGF-1 in boys with early JIA, which argues for sex-related differences in biological mechanisms involved in the disease pathogenesis.

## 1. Introduction

The term juvenile idiopathic arthritis (JIA) comprises a number of chronic inflammatory disorders with onset before 16 years of age with symptoms presenting for longer than six weeks. JIA is the most common rheumatic disease in children, with a prevalence from 3.8 to 400 per 100,000 in the European population [1]. There are seven clinical categories defined in the JIA spectrum according to the criteria from the International League of Associations for Rheumatology (ILAR) [2]. Distinct distributions regarding age at onset and sex of the child, as well as the course of the disease and outcome, vary between the different categories [3, 4]. The pathogenesis of JIA is currently unknown, but is thought to be due to a combination of environmental triggers and specific immunogenic factors [5].

Impaired linear growth is a commonly encountered complication in children with JIA, which can result in short final height [6, 7]. The etiology of growth retardation in JIA is not fully elucidated, but elevated levels of proinflammatory cytokines, delayed onset of puberty, malnutrition, and long-term glucocorticoid therapy have been implicated [6, 8, 9]. The growth hormone (GH)/insulin-like factor-1 (IGF-1) axis is a main regulator of linear growth [7], and the major part of circulating IGF-1 levels is liver derived [10]. In healthy children, circulating IGF-1 levels increase with age in pre- and early puberty, while in late puberty, this relationship is negative [11]. Therefore, normal IGF-1 levels vary in different age groups for boys and girls [11]. Several studies have demonstrated decreased IGF-1 levels in children with JIA compared to healthy controls [12–15]. However, as the JIA disease duration varied greatly and the numbers of JIA patients included in these studies were relatively low, sex-related differences were not examined. Thus, it remains to be elucidated if there are distinctions in IGF-1 levels between boys and girls with JIA and if IGF-1 levels differ in early disease compared to established JIA. To address these gaps in knowledge, we here examined circulating levels of IGF-1 separately in boys and girls who were newly diagnosed with JIA (≤1 month from JIA diagnosis), who had established JIA with a mean disease duration of 18 months, and in age- and sex-matched controls.

## 2. Materials and Methods

### 2.1. Patients and Healthy Controls

The patient group comprised 131 Estonian children diagnosed with JIA during the population-based prevalence and incidence studies in the Children's Clinic, Tartu University Hospital and Tallinn Children's Hospital during 1995–2000 [16–18]. The inclusion criteria to the study were as follows: children under the age of 16 years who had (a) arthritis of unknown cause in at least one joint for at least 6 weeks or (b) inflammatory back pain and enthesitis, or (c) spiking fever together with other symptoms suggestive of systemic arthritis. Arthritis was defined as a swollen joint or two of the following symptoms: (a) limitation of movement, (b) warmth/redness, and (c) pain on active or passive movements. All patients were classified according to the ILAR revised criteria for JIA into the following categories: persistent or extended oligoarthritis, rheumatoid factor- (RF-) positive polyarthritis, RF-negative polyarthritis, systemic arthritis, psoriatic arthritis, and enthesitis-related arthritis [2]. Children who did not fulfill any or fulfilled the criteria for more than one subtype were categorized as “other arthritis.” Children presenting with other explanations for arthritis, such as infection, trauma, and systemic connective tissue disease, were excluded. Children included at their visit to pediatric rheumatologist with no previous JIA diagnosis and ≤1 month from diagnosis were defined as patients with early JIA. The majority were treatment naïve for steroids (92%) and DMARDs (85%) (Table 1), or the drug was just initiated during the first weeks before inclusion. Children who were diagnosed with JIA more than one month ago and had received treatment longer than 1 month were defined as established JIA. The median symptom duration (the interval between time when the first symptoms were noticed and the time when the diagnosis was made by the pediatric rheumatologist) for early JIA group was 6 months (IQR 1–12): respectively, 5 months (1–12) for boys and 6 months (1.5–12) for girls. The baseline characteristics of early and established JIA patients including the total number of patients and the number of patients with available data are presented in Table 1. The control group comprised of 47 sex- and age-matched children with no arthritis or any other inflammatory autoimmune conditions. The study was approved by the Ethics Committee of Tartu University (173/T-5 and 200T-1) in accordance with the Declaration of Helsinki, and informed consent for participation in the study was obtained from all parents and/or children.

### 2.2. Clinical and Laboratory Assessment

Clinical examinations by a pediatric rheumatologist at the tertiary hospitals were performed for all children and disease activity variables, i.e., erythrocyte sedimentation rate (ESR), C-reactive protein (CRP), complete blood count with differential, swollen and tender joints, joints with limited range of motion, and joints painful in passive and/or active movements were recorded. CRP and rheumatoid factor (RF) were measured using turbidimetry with normal range 0–5 mg/l and <14 IU/ml, respectively. Anti-nuclear antibodies (ANA) were assessed by indirect immunofluorescence (IIF) using rat kidney-liver-stomach sections and considered positive if a titer ≥ 1 : 10 on two or more occasions at least 8 weeks apart were detected in the blood. If ANA was assessed by IIF using Hep-2 cells as a substrate, a titer ≥ 1 : 100 on two or more occasions was considered positive. The cut-off levels for both methods were established according to common laboratory routines by analyzing the healthy local population.

### 2.3. Determination of IGF-1 in Serum

Blood samples were obtained from cubital vein into the tubes without additive. Collected blood samples were centrifuged within 4 h at 1400*g* for 10 min, immediately aliquoted, and stored at −80°C until assayed. Previous studies have shown that IGF-1 levels are relatively stable up to more than 20 years if properly stored at −80°C and if repeated freeze-thaw cycles are avoided [19, 20]. The serum levels of IGF-1 were determined using a specific sandwich ELISA kit (R&D Systems, Minneapolis, MN, USA) according to the instructions of the manufacturer. The minimum detectable concentration after taking into account the dilution factors and standard curve was 0.3 ng/ml.

### 2.4. Statistical Analysis

The D'Agostino and Pearson omnibus normality test were used to assess if the data were normally distributed (GraphPad Prism, San Diego, USA). Kruskal-Wallis test followed by Dunn's multiple comparison test were used to compare IGF-1 levels between early JIA, established JIA, and controls. The effect of sex and disease on IGF-1 levels was analyzed using two-way ANOVA with the factors sex (*P*_sex_), disease (*P*_disease_), and their interaction (*P*_sex∗disease_) followed by Tukey's multiple comparisons test. Two-tailed Spearman rank correlation test was used to assess correlations between two variables. A *p* value < 0.05 was regarded as being statistically significant (^∗^*p* < 0.05, ^∗∗^*p* ≤ 0.01, and ^∗∗∗^*p* ≤ 0.001). Fisher's exact probability test was used to assess differences between groups with regard to disease characteristics. Fisher's r-to-z transformation analysis was used to compare correlation coefficients. Linear regression analysis was used to investigate the relationship between two variables (age and height in relation to IGF-1 levels). Multivariate factor analysis orthogonal projection to latent structures (OPLS), as previously described in detail [21], was implemented to investigate the relationship between IGF-1 serum levels (Y-variable) and disease activity/inflammation variables (X-variables) among girls and boys with early JIA (SIMCA-P+ software; Umetrics, Umeå, Sweden).

## 3. Results

### 3.1. Patient Characteristics

Blood samples were obtained from 131 JIA patients (mean ± SD age 10.2 ± 4.3 years, 43% boys) and from 47 sex- and age-matched controls (mean age 9.3 ± 4.1 years, 43% boys). Of the children with JIA, 65 (50%) were diagnosed with JIA at entry into the study and classified as early JIA (≤1 month since the diagnosis), whereas 66 (50%) were classified as established JIA patients (mean disease duration 17.9 ± 14.0 months). There was a lower prevalence of persistent oligoarthritis (*P* = 0.004) and a higher prevalence of enthesitis-related arthritis (*P* = 0.02) in the early JIA group compared to established JIA. Otherwise, these two groups were similar with regard to disease characteristics and inflammatory parameters at diagnosis. There were no sex-related differences in disease characteristics, including clinical categories of JIA and inflammatory parameters among the patients with early or established JIA (Table 1). The vast majority of children who were classified as early JIA patients were treatment naïve for DMARDs (85%) and steroids (92%). In the early JIA group, all children with systemic disease (1 boy, 3 girls) and 1 boy with polyarthritis initiated glucocorticoid treatment at diagnosis (8% of boys and 9% of girls). In addition, 4 boys with early JIA (15%) and 5 girls with early JIA (15%) had received hydroxychloroquine treatment for about one month before the inclusion to the study. In the established JIA group, 87% of boys and 63% of the girls received DMARDs, mainly hydroxychloroquine only or in combination with azathioprine (*n* = 2) or methotrexate (*n* = 2). None of the patients had received biologic therapy at inclusion with biologic anticytokine drugs.

### 3.2. Boys with Early JIA Present with Decreased IGF-1 Levels

**Figure 1:**
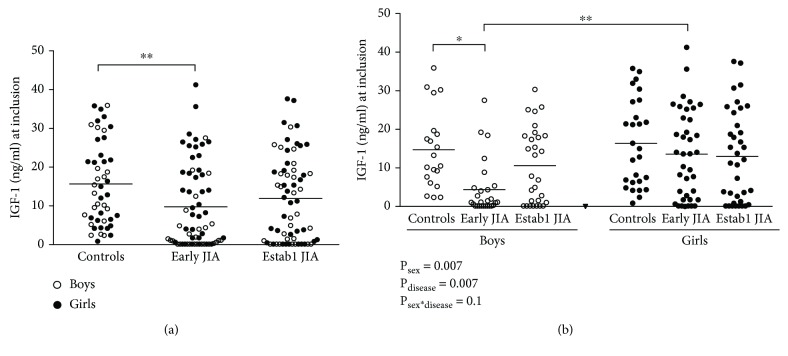
(a) IGF-1 serum levels among all control children (*n* = 47), all patients with early JIA (*n* = 65), and all patients with established JIA (*n* = 66). (b) IGF-1 serum levels among boys and girls with early JIA (*n* = 27 and *n* = 38, respectively), established JIA (*n* = 29 and *n* = 36, respectively), and in age-matched controls (*n* = 20 and *n* = 27, respectively). Horizontal bars indicate means. Data were analyzed using a two-way ANOVA with the factors sex, disease, and their interaction followed by Tukey's multiple comparisons test. ^∗^*P* ≤ 0.05 and ^∗∗^*P* ≤ 0.01.

First, we examined the levels of serum IGF-1 levels in all early JIA patients, established JIA patients, and in controls. As shown in Figure 1(a), there were significantly lower IGF-1 levels in the group of early JIA patients, but not established JIA patients, compared to controls. To examine the effect of sex and disease on IGF-1 levels, a two-way ANOVA was conducted. There was a sex- and disease-related difference in IGF-1 levels (*F*_(1,171)_ = 7.4, *P*_sex_ = 0.007; *F*_(2,171)_ = 5.2, *P*_disease_ = 0.007). However, no significant interaction between sex and disease was detected (*F*_(2,171)_ = 2.3, *P*_sex∗disease_ = 0.1). IGF-1 levels were significantly lower in boys with early JIA compared to age-matched controls, while girls displayed similar IGF-1 levels between the three groups (Figure 1(a)). The IGF-1 levels were 12-fold lower in boys compared to girls with early JIA, but no such sex-related differences were observed among controls or patients with established JIA.

### 3.3. The IGF-1 System Is Disturbed in Early JIA

**Figure 2:**
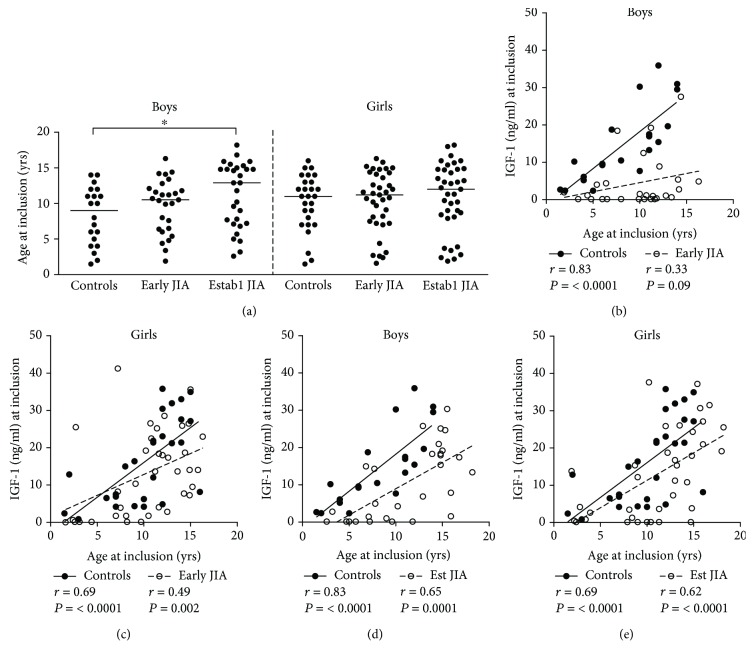
(a) Age of boys and girls at inclusion to the study. The horizontal bars indicate the median. (b, c) Correlations between serum IGF-1 levels and age in control groups of boys and girls, respectively (solid symbols), and in boys and girls with early JIA (open symbols). (d, e) Correlations between serum IGF-1 levels and age in control groups of boys and girls, respectively (solid symbols), and between boys and girls with established JIA (open symbols) (Spearman rank correlation test). (b–e) The regression lines are presented in the correlations plots. ^∗^*P* ≤ 0.05 (Kruskal-Wallis test followed by Dunn's multiple comparison test).

Age could be a potential confounding factor for the observed differences in IGF-1 levels among boys with or without early JIA. In multiple regression analysis, age contributed independently to the IGF-1 levels in both boys and girls (*P* ≤ 0.0001 for both sexes). However, as the median age at inclusion of boys with early JIA did not differ significantly from the other two groups (Figure 2(a)), decreased IGF-1 in boys with early JIA cannot solely be explained by age.

Next, we examined how IGF-1 levels were related to age in the different subgroups. Age correlated strongly to IGF-1 in serum among male controls, but not in boys with early JIA (*r* = 0.83 vs. *r* = 0.33, respectively) (Figure 2(b)). Both the correlation coefficients and the regression slopes differed significantly for these two independent groups (*P* = 0.009 and *P* = 0.006, respectively). Similarly to boys, IGF-1 levels correlated strongly to age in female controls (Figure 2(c)). In contrast to boys, there was a moderate correlation between age and IGF-1 in girls with early JIA (Figure 2(c)). Accordingly, the correlation coefficients (*r* = 0.69 vs *r* = 0.49, *P* = 0.23) and the regression slopes (*P* = 0.23) were similar in controls and early JIA girls. In established JIA, there was a strong correlation between age and serum IGF-1 levels in both sexes (Figures 2(d) and 2(e)).

**Figure 3:**
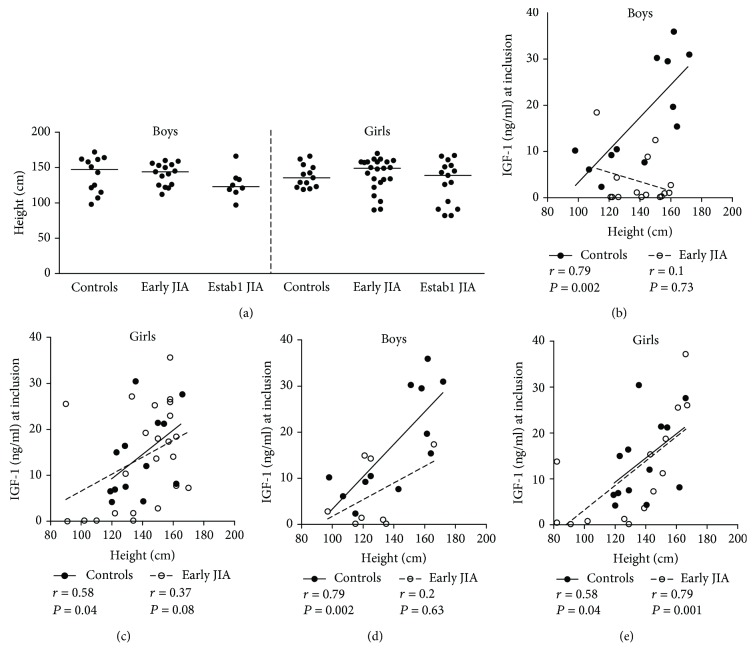
(a) Height of boys and girls at inclusion to the study. The horizontal bars indicate the median. (b, c) Correlations between serum IGF-1 levels and height in control groups of boys and girls, respectively (solid symbols), and in boys and girls with early JIA (open symbols). (d–e) Correlations between serum IGF-1 levels and height in control groups of boys and girls, respectively (solid symbols), and between boys and girls with established JIA (open symbols) (Spearman rank correlation test). (b–e) The regression lines are presented in the correlation plots.

There were no significant differences in height between the groups of children, either among boys or girls (Figure 3(a)), but boys with established JIA had tendency towards shorter body height. Similar to age, height correlated strongly to serum IGF-1 in male controls (*r* = 0.79), but not in those with early JIA (Figure 3(b)), and the correlation coefficients differed significantly (*P* = 0.03) between the groups as well as the regression slopes (*P* = 0.003) (Figure 3(b)). Among girls, height correlated to serum IGF-1 in controls and there was a trend for correlation also in early JIA (Figure 3(c)). Neither the correlation coefficients (*P* = 0.5) nor the regression slopes (*P* = 0.7) differed between control group of girls and in girls with early JIA. In established JIA, there was a significant correlation between height and serum IGF-1 levels in girls but not in boys (Figures 3(d) and 3(e)). Overall, these results indicate a disturbance in the IGF-1 system in relation to age and anthropometrics in the early JIA, especially among boys.

### 3.4. IGF-1 Levels in Relation to Clinical Disease Activity

**Figure 4:**
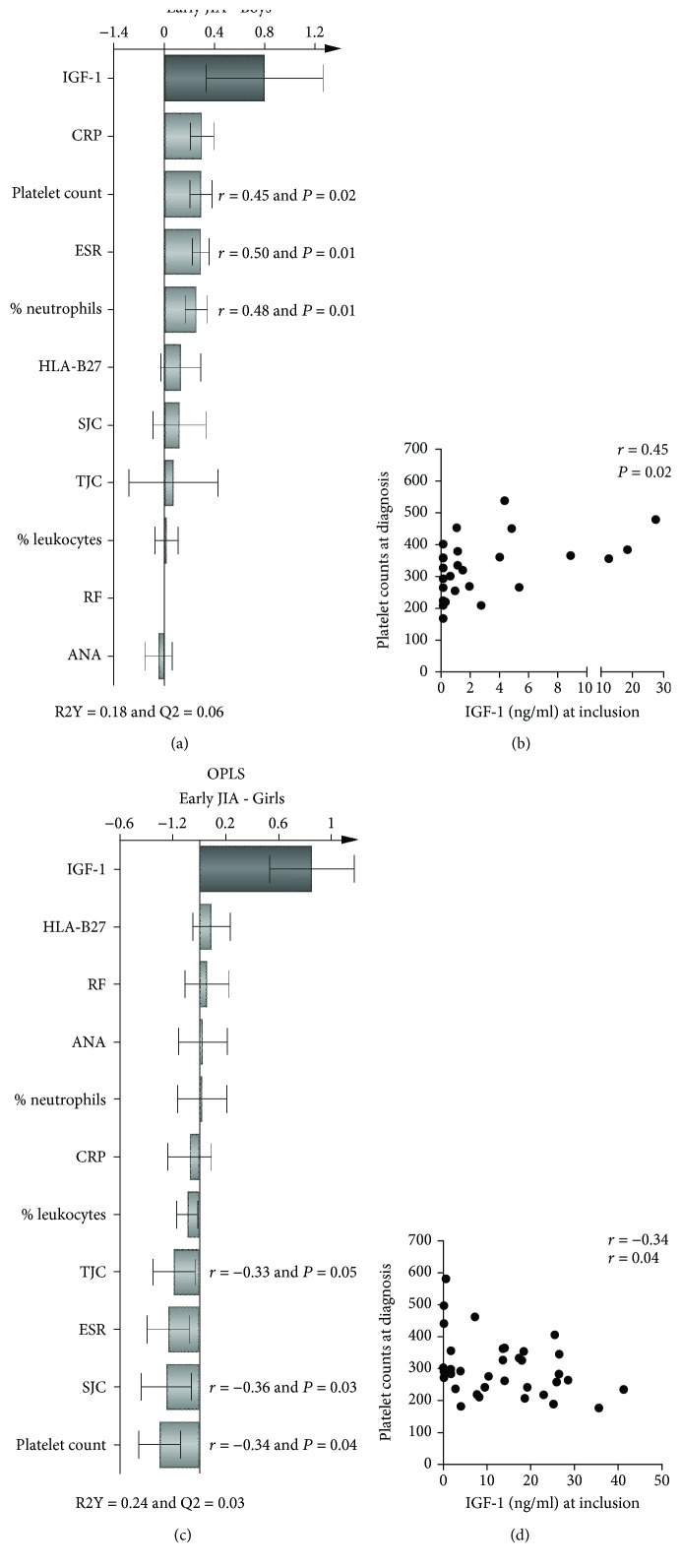
Serum IGF-1 levels in relation to disease activity/inflammation variables among boys and girls with early JIA. Multivariate OPLS loading column plots displaying the associations between Y, i.e., IGF-1 serum levels, and X-variables, i.e., clinical disease activity/inflammation variables, among (a) boys and (c) girls with early JIA. X-variables with bars projected in the same direction as Y are positively associated, whereas parameters in the opposite direction are inversely related to Y. The larger the bar and smaller the error bar, the stronger and more certain is the contribution to the model. Correlations between serum IGF-1 levels and platelet counts in boys (b) and in girls (d). (b, d) Spearman's rank correlation test.

We next investigated whether serum IGF-1 was associated with disease-related variables in early JIA by the use of multivariate factor analysis. As shown in the OPLS loading column plot in Figure 4(a), higher IGF-1 among boys were positively related to higher CRP and correlated significantly to platelet counts (Figure 4(b)), ESR, and proportion of neutrophils. Among girls, higher IGF-1 levels correlated negatively with platelet counts, swollen joint counts, and tender joint counts (Figures 4(c) and 4(d)). The presence of RF, ANA, and HLA-B27 were not related to IGF-1 levels in either boys or girls (Figures 4(a) and 4(b)).

## 4. Discussion

Decreased serum IGF-1 levels are a common feature among children with JIA. The main novel findings observed in the present study were (a) boys with early JIA have almost 12-fold lower serum IGF-1 levels compared to age-matched girls with early JIA and (b) boys with early JIA have significantly lower IGF-1 levels relative to age-matched male controls.

One possible explanation for lower IGF-1 levels among boys compared to girls with early JIA could be differences in their pubertal age. On average, boys usually begin puberty at an older age than girls. In healthy children, circulating IGF-1 levels are positively related to age in prepuberty (mean age: 9.4 years for boys and 8.5 years for girls) and early puberty (mean age: 13.3 years for boys and 12.1 years for girls), while the relationship between these two factors is negative in late puberty [11]. The majority of the boys in our early JIA group were in prepuberty whereas the girls were already in early or midpuberty [11]. Increased estrogen levels could be expected in our early/midpuberty study girls as compared to their prepubertal period. Rising estrogen (estradiol) levels transiently increase growth hormone (GH) concentrations, which in turn lead to increase in IGF-1 levels. Additionally, serum IGF-1 levels have been found to increase in parallel with the transient rise in insulin resistance that occurs during pubertal development [22, 23].

In line with our findings, low serum IGF-1 levels have previously been reported in several studies of JIA [12–15], but IGF-1 levels in the normal range have also been found [24]. However, these studies consisted of relatively small JIA cohorts, most of them including less than 25 children, and male and female patients were pooled. The disease duration for the included JIA patients varied considerably from a few months up to several years, children with different treatments were analyzed together, and early JIA was not specifically investigated. An important strength with our JIA cohort is the relatively large number of included patients, which enabled separate analyses of IGF-1 levels in boys and girls as well as distinctions between early and established JIA.

We also observed that boys with early JIA displayed significantly lower IGF-1 serum levels compared to age-matched male controls. One possible explanation for this finding could be delayed onset of puberty compared to healthy children (reviewed in [7]). In this study, samples from population-based prevalence and incidence cohorts of JIA were utilized and since these studies had other epidemiological aims, pubertal development data were unfortunately not recorded. This is an important limitation of our study since pubertal development influences serum IGF-1 levels and therefore may affect comparison between age-matched controls and children with JIA as well as between sexes. However, this does not change our intriguing observation that IGF-1 levels in early JIA boys were significantly lower.

In children with systemic and polyarticular JIA, growth failure is a common feature [1, 7, 25–27]. Several studies also demonstrate that slow growing children with JIA present with significantly lower serum IGF-1 levels compared to those in healthy controls [13, 14, 28]. IGF-1 deficiency could have severe clinical consequences in children and lead to growth failure resulting in short adult height [29, 30]. In the present study, age and height correlated strongly to serum IGF-1 levels in the control groups, but not in boys with early JIA. Our findings could indicate that there is a potential dysregulation of IGF-1 production in the pathogenesis of early JIA among boys. However, our results should be interpreted with some caution since all data regarding height and weight were not available.

Several studies have demonstrated interaction between IGF-1 and proinflammatory cytokines, which are commonly elevated in JIA patients [8, 31, 32]. Inflammation-related cytokines, including TNF-*α*, IL-1*β*, and IL-6, have been shown to dysregulate IGF-1 downstream intracellular signaling in chondrocytes [33–35]. Moreover, elevated IL-6 serum levels were associated with low circulating IGF-1 levels and growth delay in a transgenic mouse model [36], and serum IL-6 was inversely correlated with IGF-1 levels in children with systemic JIA [32, 36, 37]. Also, systemic JIA patients who were treated with anti-IL-6 receptor antibody (tocilizumab) experienced a catch-up in growth and an increase in serum IGF-1 levels [38]. In the present population-based JIA cohort, the majority of the established JIA children received nonbiological antirheumatic treatment with DMARDs, while most early JIA patients were treatment-naïve, which could explain normalized IGF-1 levels in established disease.

Few studies have examined serum IGF-1 levels in relation to inflammation- and disease activity-related variables in JIA. In a JIA cohort including all categories, but systemic JIA, serum IGF-1 levels correlated inversely to CRP [24]. In another cohort including systemic JIA patients only, there was also a negative association, although not statistically significant, between IGF-1 levels and CRP [38]. In both of these cohorts, boys and girls were analyzed together. In the present study, we observed an opposite pattern of association with disease activity measurements between male and female patients with early JIA. In boys, higher IGF-1 levels were related positively to inflammation-related markers, whereas they were either negatively associated or unrelated in girls. The underlying mechanisms for these sex-based discrepancies are unclear.

## 5. Conclusions

We here report for the first time that boys, but not girls, with early JIA present with decreased IGF-1 levels. Despite accumulating evidence that support sex-related differences in immune responses and in the prevalence of autoimmune diseases [39], a majority of JIA studies do not analyze data by sex. Based on our results, there is a need for further investigation of sex-based differences in biological mechanisms involved in the very early phase of JIA process in larger cohorts.

## Figures and Tables

**Table 1:** Clinical and demographic characteristics of boys and girls with early JIA (≤1 month from diagnosis) and established disease (>1 month from diagnosis).

	Early JIA all (*n* = 65)	Establ JIA all (*n* = 66)	Early JIA boys (*n* = 27)	Early JIA girls (*n* = 38)	Establ JIA boys (*n* = 29)	Establ JIA girls (*n* = 37)
Age at inclusion, years (mean ± SD)	9.7 ± 4.0	10.8 ± 4.6	9.3 ± 3.8	10.0 ± 4.2	10.8 ± 4.6	10.7 ± 4.6
Age at diagnosis, years (mean ± SD)	9.7 ± 4.0	9.4 ± 4.3	9.3 ± 3.8	10.0 ± 4.2	9.3 ± 4.1	9.1 ± 4.6
Disease duration, months (mean ± SD)	0.06 ± 0.2	17.9 ± 14.0^∗∗∗^	0.0 ± 0	0.1 ± 0.3	19.2 ± 13.7	16.9 ± 14.4
DMARDs, *n* (%)^§^	9 of 60 (15)	26/35 (74)^∗∗∗^	4/26 (15)	5/34 (15)	14/16 (87)	12/19 (63)
Steroids, *n* (%)^§^	5 of 60 (8)	6/35 (17)	2/26 (8)	3/34 (9)	1/16 (6)	5/19 (26)
ANA-positive, *n* (%) ^§^	16 of 50 (32)	6/21 (29)	3/19 (16)	13/31 (42)	5/11 (45)	1/10 (10)
HLAB27-positive, *n* (%) ^§^	18 of 62 (29)	20/63 (32)	10/26 (38)	8/36 (22)	8/28 (29)	11/34 (32)
RF-positive, *n* (%)	2 of 51 (4)	3/29 (10)	0/21 (0)	2/30 (7)	1/15 (7)	2/14 (14)
SJC, *n* (median, IQR)	1 (1–3)	1 (0–2.5)	2 (1–4)	1 (0.25–2.75)	1.5 (0–3.5)	1 (0–2.5)
TJC, *n* (median, IQR)	1 (0–1)	1 (0–1.75)	1 (0–1)	0.5 (0–1)	0 (0–0)	0 (0–2)
ESR at diagnosis, mm/h (median, IQR)	15 (7–29)	11 (6–25)	13 (8–32)	15 (7–28)	15 (7–26)	7 (5–21)
CRP mg/l, median (IQR) ^#^	39 (30–63)	48 (18–115)	42 (26–59)	38 (32–128)	80 (18–130)	33 (22–133)
Neutrophils at diag, % (median, IQR)	53 (46–62)	60 (49–67)	51 (46–66)	54 (44–62)	61 (48–69)	59 (48–66)
JIA categories, *n* (%)						
(i) Oligoarthritis, persistent	16 (25)	33 (50)^∗∗^	7 (26)	9 (24)	18 (62)	15 (41)
(ii) Oligoarthritis, extended	11 (17)	8 (12)	3 (11)	8 (21)	2 (7)	6 (16)
(iii) Polyarthritis, RF-positive	2 (3)	3 (5)	0	2 (5)	1 (3)	2 (5)
(iv) Polyarthritis, RF-negative	15 (23)	16 (24)	5 (19)	10 (26)	4 (14)	12 (32)
(v) Enthesitis-related	8 (12)	1 (2)^∗^	6 (22)	2 (5)	1 (3)	0
(vi) Systemic	4 (6)	3 (5)	1 (4)	3 (8)	3 (10)	0
(vii) Psoriatic	4 (6)	0 (0)	2 (7)	2 (5)	0	0
(viii) Other	5 (8)	1 (2) †	3 (11)	2 (5)	0	2 (5) †

IQR: interquartile range. ^§^Data for ANA, HLAB27, and treatment not available for all children in the cohort. ^#^Median CRP values shown for children with increased CRP. †Category missing for one patient. Statistical comparisons between all early JIA vs. all established JIA: ^∗∗∗^*P* < 0.0001, ^∗∗^*P* < 0.005, and ^∗^*P* < 0.05.

## Data Availability

The data used to support the findings of this study are available from the corresponding author upon reasonable request.
